# Successful Bailout for Transcarotid Delivery Failure of Transcatheter Aortic Valve

**DOI:** 10.1016/j.jaccas.2026.108920

**Published:** 2026-06-18

**Authors:** Keisuke Miyamoto, Tomohiro Suenaga, Kenichi Ishizu, Shinichi Shirai, Kenji Ando

**Affiliations:** Department of Cardiology, Kokura Memorial Hospital, Kitakyushu, Japan

**Keywords:** buddy balloon technique, TC-TAVI

## Abstract

**Case Summary:**

Transcarotid–transcatheter aortic valve implantation (TC-TAVI) generally enables smooth transcatheter heart valve crossing given its proximity to the aortic annulus; however, severe anatomical constraints may still lead to delivery failure. We report a TC-TAVI case in which transcatheter heart valve passage across the aortic valve was initially unsuccessful because of aortic angulation and extensive leaflet calcification. This technical difficulty was effectively resolved using the buddy balloon technique, which enabled successful valve advancement. This case demonstrates that the superior pushability of the TC approach may not overcome extreme anatomical resistance, necessitating predilatation aggressively indicated for such challenging anatomies.

**Take-Home Messages:**

Pronounced aortic angulation and severe leaflet calcification can hinder transcatheter heart valve delivery, even during TC-TAVI, where easier valve crossing is typically anticipated. These anatomical factors remain significant predictors of delivery failure, irrespective of the vascular access route.

## Graphical Abstract

**Figure:**
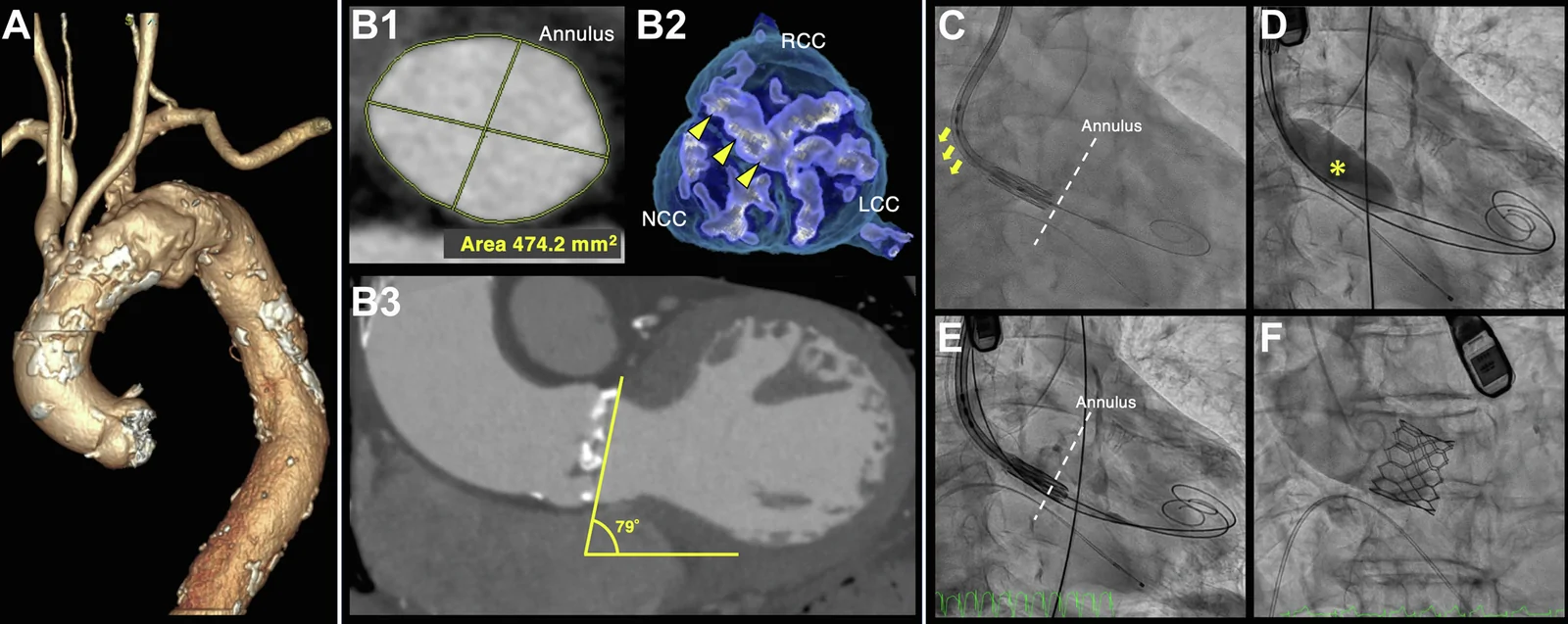

A 93-year-old man with symptomatic severe aortic stenosis was referred for transcatheter aortic valve implantation (TAVI). Considering the presence of a diffusely atherosclerotic descending aorta (Figure 1A) and narrow iliofemoral arteries (minimum diameter: 4.4 mm), a right transcarotid (TC) approach was selected after confirming adequate intracranial collateralization and a right common carotid artery diameter of 7.0 mm. Preprocedural computed tomography revealed right coronary cusp–dominant bulky calcification of the aortic valve leaflets with an Agatston score of 3,474 Hounsfield units as well as marked horizontal aortic angulation of 79°, which was measured between the horizontal and aortic annular planes (Figure 1B).

**Figure 1:**
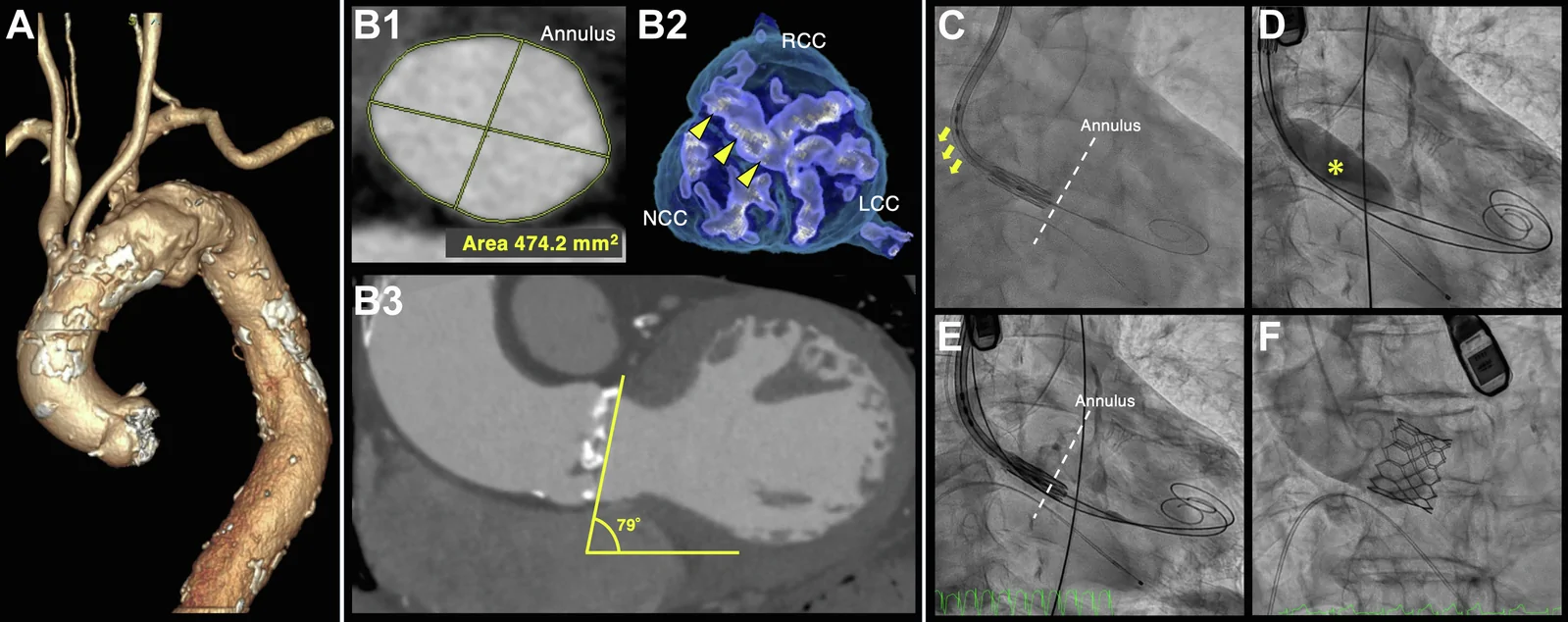
Anatomical Challenges and Buddy Balloon Bailout in Transcarotid–Transcatheter Aortic Valve Implantation Preprocedural computed tomography demonstrating (A) shaggy plaques on the descending aorta and aortic arch and (B1 to B3) a marked horizontal aortic angulation (79°) and RCC-dominant bulky calcification (yellow arrowheads) on aortic valve leaflets, with an annulus area of 474.2 mm^2^. (C to F) A series of fluoroscopic images illustrating (C) failed THV advancement across the aortic valve with pushing force deviating delivery shaft toward the greater curvature of the aorta (yellow arrows), (D) inflation of the buddy balloon (asterisk), (E) successful THV advancement immediately after deflation of the buddy balloon, and (F) final aortic angiogram after successful deployment of the THV. LCC = left coronary cusp; NCC = noncoronary cusp; RCC = right coronary cusp; THV = transcatheter heart valve.

After surgical exposure of the right common carotid artery, a Safari S guidewire (Boston Scientific) was advanced across the native aortic valve. Direct implantation of a 26-mm SAPIEN 3 Ultra RESILIA (Edwards Lifesciences) transcatheter heart valve (THV) without predilatation was attempted. However, the THV failed to cross the aortic valve (Figure 1C and Video 1). During repeated advancement attempts, the patient developed hemodynamic instability, necessitating an immediate bailout strategy. A 7-F sheath was urgently inserted via the right femoral artery, and a second Safari S wire was advanced into the left ventricle to function as a buddy wire, followed by balloon aortic valvuloplasty using an 18-mm TRIVAL balloon (Kaneka Medix) (Figure 1D). The THV was advanced simultaneously during the deflation of the TRIVAL balloon while it remained in a transvalvular position, resulting in successful THV crossing (Figure 1E and Video 2). After confirming removal of the second left ventricular wire, THV implantation was completed (Figure 1F). Postprocedural echocardiography demonstrated optimal THV positioning without paravalvular leakage. The postoperative course was uneventful, and the patient was discharged without neurological complications.

In general, advancing a balloon-expandable THV delivery system across the aortic valve is straightforward; however, extreme aortic angulation and severe leaflet calcification are well recognized as risk factors for device delivery failure.^1^ Flex adjustment, while effective in transfemoral TAVI, has limited steerability in the short trajectory of the TC approach. Several bailout techniques, including the buddy balloon, “chaperone” technique, and slight balloon inflation (eg, 0.5 mL), remain less well described in alternative access routes.^1^, ^2^, ^3^ Additionally, pre-emptive use of a large-bore sheath (eg, 24-F to 26-F) can facilitate THV retrieval and subsequent balloon dilatation, if vessel diameter permits.

In this case, we encountered critical balloon-expandable THV delivery failure during TC-TAVI, accompanied by hemodynamic instability, which was successfully managed using the buddy balloon technique. Although the TC approach is theoretically associated with enhanced pushability given its proximity to the aortic valve, this case underscores that extreme aortic angulation and severe leaflet calcification can potentially cause device delivery failure, even when using an access route that facilitates more direct force transmission. In addition, given the trajectory of a right TC approach, heavy calcification of the right coronary cusp and noncoronary cusp may have contributed to impeding the device delivery to a greater extent compared with the transfemoral approach. In other words, the TC approach is not a guaranteed ticket to skip predilatation, and a lower threshold for predilatation should be considered in patients with these potentially high-risk anatomical features.

## Funding Support and Author Disclosures

Dr Ishizu is a proctor of intracardiac echocardiography-guided TAVI for Abbott Medical; and has served as the screening proctor for Edwards Lifesciences. Dr Shirai is a proctor of transfemoral TAVI for Edwards Lifesciences, Medtronic, and Abbott Medical. All other authors have reported that they have no relationships relevant to the contents of this paper to disclose.

**Take-Home Messages**

• Pronounced aortic angulation and severe leaflet calcification can hinder transcatheter heart valve delivery, even during transcarotid–transcatheter aortic valve implantation, where easier valve crossing is typically anticipated.
• These anatomical factors remain significant predictors of delivery failure, irrespective of the vascular access route.

## Appendix

Video 1 Failed Transcatheter Heart Valve Crossing During Transcarotid–Transcatheter Aortic Valve Implantation

Video 2 Buddy Balloon Bailout Facilitating Successful Valve Crossing
